# Reemergence of Dengue Virus Serotype 3, Brazil, 2023

**DOI:** 10.3201/eid2907.230595

**Published:** 2023-07

**Authors:** Felipe Gomes Naveca, Gilberto A. Santiago, Rodrigo Melo Maito, Cátia Alexandra Ribeiro Meneses, Valdinete Alves do Nascimento, Victor Costa de Souza, Fernanda Oliveira do Nascimento, Dejanane Silva, Matilde Mejía, Luciana Gonçalves, Regina Maria Pinto de Figueiredo, Ana Cecília Ribeiro Cruz, Bruno Tardelli Diniz Nunes, Mayra Marinho Presibella, Nelson Fernando Quallio Marques, Irina Nastassja Riediger, Marcos César Lima de Mendonça, Fernanda de Bruycker-Nogueira, Patricia C Sequeira, Ana Maria Bispo de Filippis, Paola Resende, Tulio Campos, Gabriel Luz Wallau, Tiago Gräf, Edson Delatorre, Edgar Kopp, Andrea Morrison, Jorge L. Muñoz-Jordán, Gonzalo Bello

**Affiliations:** Fundação Oswaldo Cruz, Rio de Janeiro, Brazil (F.G. Naveca, M.C.L. Mendonça, F. de Bruycker-Nogueira, P.C. Sequeira, A.M.B. de Filippis, P. Resende, G. Bello);; Centers for Disease Control and Prevention, San Juan, Puerto Rico, USA (G.A. Santiago, J.L. Muñoz-Jordán);; Laboratório Central de Saúde Pública de Roraima, Boa Vista, Brazil (R.M. Maito, C.A.R. Meneses);; Fundação Oswaldo Cruz, Manaus, Brazil (F.G. Naveca, V.A. Nascimento, V.C. Souza, F.O. do Nascimento, D. Silva, M. Mejía);; Fundação de Vigilância em Saúde–Dra. Rosemary Costa Pinto, Manaus (L. Gonçalves);; Fundação de Medicina Tropical–Dr Heitor Vieira Dourado, Manaus (R.M.P. Figueiredo);; Seção de Arbovirologia, Instituto Evandro Chagas, Ananindeua, Brazil (A.C.R. Cruz, B.T.D. Nunes);; Laboratório Central de Saúde Pública do Paraná, Curitiba, Brazil (M.M. Presibella, N.F.Q. Marques, I.N. Riediger);; Fundação Oswaldo Cruz, Recife, Brazil (T. Campos, G.L. Wallau);; Bernhard Nocht Institute for Tropical Medicine Department of Arbovirology, Hamburg, Germany (G.L. Wallau);; Fundação Oswaldo Cruz, Curitiba (T. Gräf);; Universidade Federal do Espírito Santo, Alegre, Brazil (E. Delatorre);; Florida Department of Health, Tampa, Florida, USA (E. Kopp, A. Morrison)

**Keywords:** dengue, viruses, arboviruses, mosquito-borne diseases, vector-borne diseases, Brazil

## Abstract

We characterized 3 autochthonous dengue virus serotype 3 cases and 1 imported case from 2 states in the North and South Regions of Brazil, 15 years after Brazil's last outbreak involving this serotype. We also identified a new Asian lineage recently introduced into the Americas, raising concerns about future outbreaks.

Dengue virus serotype 3 (DENV-3) consists of 5 distinct genotypes (I–V). Genotype III (GIII) is the most widespread and was associated with large outbreaks in Asia, Africa, and the Americas (*1*). DENV-3 GIII probably emerged in the Indian subcontinent around the mid-1970s and was first introduced into the Americas in the 1990s. This introduction established an endemic lineage that evolved separately from the Asian lineage for >20 years (GIII-American-I lineage) and has been extensively transmitted across the continent in subsequent years (*2*). The most recent DENV-3 GIII-American-I lineage sequences were reported in Mexico in 2021 (GenBank accession nos. OM417340 and OM417341).

The first autochthonous case of DENV-3 GIII-American-I lineage in Brazil was reported in December 2000 in Rio de Janeiro state (Southeast Region) (*3*,*4*). This lineage caused a massive dengue outbreak in Rio de Janeiro state in 2002, and subsequent outbreaks were reported in Brazil throughout the 2000s, highlighting its rapid spread (*5*). Multiple importations of the GIII-American-I lineage from the Lesser Antilles (Caribbean) into Brazil occurred through the 2000s. The Southeast and North regions of Brazil were the most important dissemination hubs (*6*,*7*). In contrast, public data reveals that DENV-3 represented a small fraction (<1%) of total dengue cases in Brazil since 2010 (Appendix Figure), and few cases have been confirmed by sequencing. Thus, DENV-3 transmission has not been reported for the last few years, suggesting that the DENV-3 GIII-American-I lineage may have become extinct in Brazil.

We describe the genetic characterization of 4 DENV-3 cases detected in Brazil in 2023. Three autochthonous cases were detected in Roraima State by the Roraima State Central Laboratory during January 3–March 4, and 1 case imported from Suriname was detected in Paraná State by the Paraná State Central Laboratory on March 12. Serum samples were sent for sequencing at Fundação Oswaldo Cruz Amazônia (Manaus, Brazil), part of the Fundação Oswaldo Cruz Genomics Surveillance Network Consortium of Brazil’s Ministry of Health. We obtained 4 complete DENV-3 genomes by using the Viral Surveillance Panel (Illumina, https://www.illumina.com), aligned them with DENV-3 sequences sampled worldwide, and analyzed them for genotyping and spatiotemporal diffusion reconstruction (Appendix).

We classified the 4 complete DENV-3 genomes detected in Brazil in 2023 as GIII according to the Flavivirus Genotyping Tool (https://www.rivm.nl/mpf/typingtool/flavivirus). Phylogenetic analysis of the DENV-3 GIII dataset revealed that the new GIII sequences detected in Brazil branched together with sequences sampled in Puerto Rico and Florida (USA) in 2022 from both autochthonous cases and cases imported from Cuba (Figure, panel A). The new monophyletic clade consisting of DENV-3 sequences detected in the Americas during 2022–2023 (GIII-American-II lineage) nested among sequences sampled in Asia belonging to the Asian lineage of GIII over the last decade and outside the GIII-American-I lineage that consists of sequences sampled in the Americas during 1994–2021 (Figure, panel A). Thus, the identified GIII-American-II lineage was not a reemergence of the GIII-American-I lineage previously established in the continent but a new introduction of GIII from Asia. The dissemination of a new DENV-3 lineage in a large and populous country like Brazil is concerning because many inhabitants may lack immunity against this serotype. Brazil has not faced recent outbreaks by this serotype; therefore, there is an increased risk for epidemics. Moreover, the endemicity of other DENV serotypes may increase the likelihood of an upsurge of severe cases.

**Figure:**
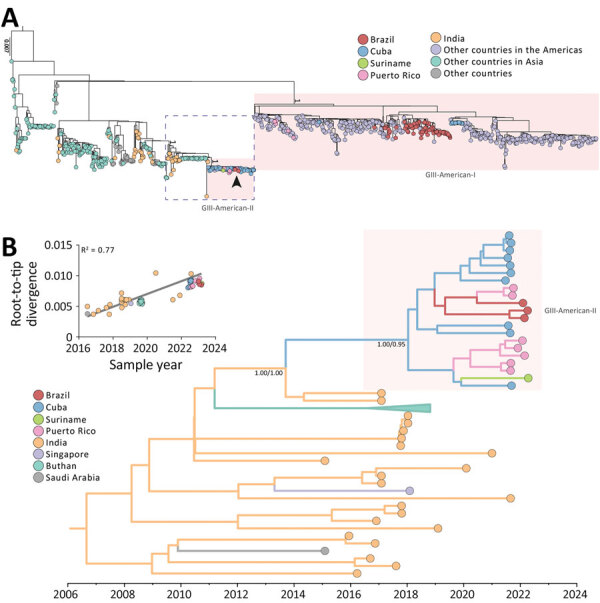
Evolutionary analysis for dengue virus serotype 3 (DENV-3) genotype III, Brazil. A) Rooted tree shows the evolutionary relationships of the complete genome sequences from the DENV-3 genotype III identified in Roraima state, Brazil (arrow), and 979 publicly available sequences from GenBank. Colors represent different sampling locations. Red highlighting shows the 2 DENV-3 GIII American lineages. The dashed blue rectangle indicates the area enlarged in panel B. Scale bar indicates nucleotide substitutions per site. B) Time-resolved maximum clade credibility tree showing the enlarged area from panel A. Colors indicate geographic sampling location. To improve visualization, the monophyletic clade for Bhutan has been collapsed. Posterior probability/posterior state probability values at key nodes show support for branching structure. Inset shows the plot of the root-to-tip genetic distance against sampling time.

The phylogeographic tree estimated for a subset of GIII-American-II sequences and the most closely related GIII Asian sequences (Appendix) indicates that this new American lineage was most probably introduced from the Indian subcontinent (posterior state probability 1) in 2019 (Bayesian credible interval 2018–2020) and infers that it could have circulated cryptically for ≈3 years before being detected in 2022 (Figure, panel B). Our phylogeographic analysis points to Cuba as the most probable (posterior state probability 0.95) introduction point of the GIII-American-II lineage (Figure, panel B). However, this result is probably biased by the absence of DENV-3 genomes representative of other Caribbean countries. Of note, the estimated onset date of the new GIII-American-II lineage coincides with a severe DENV-3 epidemic described in Jamaica during 2018–2019, the largest dengue outbreak reported on this Caribbean island in 40 years (*8*). Those observations support the hypothesis that the DENV-3 GIII-American-II lineage might have been introduced from India into the Caribbean Region around 2018–2019 and was more recently disseminated from the Caribbean to Brazil, Suriname, and Florida during 2022–2023.

In conclusion, our findings confirm the detection of DENV-3 GIII in the Northern Region of Brazil in 2023, associated with a new introduction of this genotype from the Indian subcontinent into the Americas. The >15-year absence and lack of widespread recent transmission of DENV-3 in Brazil might have rendered the population highly susceptible to infection by this serotype, highlighting the importance of early detection and the need for continuous monitoring of DENV-3 spread in Brazil and other countries in the Americas.

Appendix Additional information about reemergence of dengue virus serotype 3, Brazil, 2023.
