# Supplementary material for: Reemergence of Dengue Virus Serotype 3, Brazil, 2023
Source: Emerg Infect Dis. 2023 Jul;29(7):1482–4. doi: 10.3201/eid2907.230595 (PMC10310385; doi:10.3201/eid2907.230595)
Supplement: Appendix — Additional information about reemergence of dengue virus serotype 3, Brazil, 2023. [file 23-0595-Techapp-s1.pdf]

Article DOI: https://doi.org/10.3201/eid2907.230595

EID cannot ensure accessibility for supplementary materials supplied by authors. Readers who have difficulty accessing supplementary content should contact the authors for assistance.

Reemergence of Dengue Virus Serotype 3, Brazil, 2023

Appendix

Materials and Methods

Ethics statement, sample collection, and arboviral detection as a surveillance routine in Central State Laboratories

In Roraima State and Paraná State most arboviral suspected cases are investigated for Zika, Dengue, and Chikungunya (ZDC) by the State's Central Laboratory (LACEN-RR in Roraima and LACEN-PR in Paraná) as a routine. Between 1st January and 27th March, 319 suspected acute arboviral samples were sent to the Roraima State Central Laboratory (LACEN-RR) for molecular diagnosis. At LACEN-RR, these samples underwent a nucleic acid extraction with commercial kits, followed by ZDC detection using the reverse transcription real-time PCR kit developed by Instituto de Biologia Molecular do Paraná (IBMP - Kit Biomol ZDC - https://www.ibmp.org.br), which not only detects DENV but also identifies its serotype. Only 10 cases were confirmed as dengue (DENV-2 = 4, DENV-3 = 4) or chikungunya (n = 2) by real-time reverse transcription PCR. Clinical and epidemiologic data, plus IgM detection, confirmed 19 other cases (DENV = 9, Chikungunya = 10). The socio-demographic and laboratory characteristics of the three DENV-3 cases are summarized in supplemental Table 1. The case from Paraná described in the present manuscript is considered as an imported case from Suriname since the patient arrived in Brazil with dengue symptoms 3 days before the sample collection. This study was approved by the Ethics Committee of Instituto Oswaldo Cruz, which waived signed informed consent (CAAE: 90249218.6.1001.5248). Access to the Brazilian genetic heritage was registered under the number SISGEN # A2E0307.

Epidemiologic data collection and visualization

Epidemiologic data on dengue fever cases in Brazil were downloaded from the DATASUS database using the R package microdatasus (*1*). Specifically, data on the number of confirmed DENV cases and their corresponding serotypes were obtained. However, serotype information was only available from 2007 onwards. The data were extracted at the national level and stratified by year. Plots were produced using the ggplot2 package on R software version 4.1.2.

Nucleotide sequencing library preparation

As requested by local health authorities, DENV-3 positive serum samples were sent to Instituto Leônidas and Maria Deane (ILMD - Fiocruz Amazônia) for nucleotide sequencing and genomics characterization. At ILMD, samples were submitted to viral total nucleic acid extraction with a commercial kit (Promega Maxwell), according to the manufacturer's instructions. Thus, the total nucleic acid was used for the sequencing library with Illumina's Viral Surveillance Panel (VSP w ILMN RNA Prep w Enrich). The VSP manual (# 1000000124435 v03, April 2021) describes the preparation of viral genomic libraries through a hybrid capture method using biotinylated probes and enrichment for the complete genome sequencing of 66 human pathogens, including DENV-3, allowing the detection of mutations and new lineages, without bias.

Whole-genome sequencing and consensus genomes

The VSP libraries were then submitted to nucleotide sequencing on a MiSeq instrument using V3 cartridges on 2 × 150 cycles paired-end run (Illumina). The FastQ files generated at Illumina's cloud (https://basespace.illumina.com) were submitted to further quality check and duplicate removal steps using a customized workflow employing BBDuk (v38.84). The consensus genomes were assembled into contigs using BBMap and the GenBank DENV-3 reference genome (NC_001475) as the template. We used the BBTools (BBDuk and BBMap v38.84 - https://sourceforge.net/projects/bbmap/) versions embedded in Geneious Prime 2023.0.4 software (https://www.geneious.com). Finally, all contigs were carefully visually inspected before further analysis. The obtained consensus genomes ranged in length from 10,511 to 10,697 nt, or 98.2 to 99.9% of the DENV-3 RefSeq, with no ambiguous or unidentified sites (“N”).

DENV-3 genotype identification

The DENV-3 consensus genomes obtained in this study were initially submitted to genotype identification using the Flavivirus Genotyping Tool Version 0.1 (https://www.rivm.nl/mpf/typingtool/flavivirus/). This analysis showed that the three genomes belong to the DENV-3 genotype III, with high bootstrap support (100.0).

DENV-3 dataset and global phylogenomics reconstruction

We downloaded all DENV-3 genomes (genome length >10K) available on GenBank (2023–03–25) using the NCBI Virus portal (https://www.ncbi.nlm.nih.gov/labs/virus/vssi/#/). Subsequently, we confirmed both the serotype (DENV-3) and the genotype (GIII) using the same Flavivirus genotyping web tool previously mentioned, followed by removing sequences named as modified nucleic acids or linked to patents. Thus, this first dataset encompasses 986 DENV-3 (GIII) genomes available at GenBank, which were aligned with the four genomes obtained in this study using MAFFT v7.490 under the automatic selection of the appropriate strategy according to data size (*2*).

The aligned dataset was used to reconstruct phylogenomic relationships by maximum likelihood (ML) using IQ-TREE multicore version 2.0.3 (*3*). All sequences passed the IQ-TREE composition test, and ModelFinder (*4*) chose the GTR+F+R4 evolutionary model according to Bayesian Information Criterion. The tree branches' support was evaluated with Shimodaira–Hasegawa approximate likelihood-ratio test (SH-aLRT) (*5*) and Ultrafast bootstrap (UFBoot) (*6*), with 2,000 replicates.

Spatiotemporal and viral diffusion reconstruction of DENV-3

To reconstruct the geographic and temporal origin of DENV-3 cases in Brazil in 2023, we employed a Bayesian phylogeographic inference approach implemented in BEAST v1.10.4 (*7*). To reduce computation time in the phylogeographic reconstructions, we selected the sub-set of most closely related Asian sequences to the newly generated DENV-3 genomes, as observed in the ML analysis. Moreover, we generate a "non-redundant" sub-set of 10 DENV-3 sequences representative of the viral diversity in imported cases from Cuba detected in Florida. To achieve this aim, sequences imported from Cuba were grouped by similarity (genetic similarity >0.985) with the CD-HIT program (*8*), and only one sequence per cluster (the oldest one) was selected. Finally, we exclude potential recombinant genomes detected by at least five out of seven

different algorithms (RDP, GENECONV, Chimaera, MaxChi, BootScan, SiScan, and 3seq) running in RDP5.34 (*9*) and temporal outlier sequences that deviated more than 1.5 interquartile ranges in the regression analysis of the root-to-tip divergence against the tip sampling time of the ML phylogenetic tree performed using TempEst v.1.5.3 (*10*). Time-scaled trees were inferred with a relaxed molecular clock model (*11*), outperforming the strict clock model in marginal likelihood estimation and a Bayesian Skyline coalescent model. The ancestral node states were reconstructed with a continuous-time Markov chain (CTMC) prior (*12*) and discrete spatial diffusion with an asymmetric substitution model to infer the migration events (*13*). Bayesian stochastic search variable selection (BSSVS) was performed to identify the significant migration routes. Two Markov Chain Monte Carlo (MCMC) chains were run for 100 million generations and combined after removing 10% burn-in. Convergence was assessed by calculating the Effective Sample Size (ESS) for all parameters using Tracer v1.7.1 (*14*). The time-scaled phylogeny was visualized using FigTree v1.4.4 (http://tree.bio.ed.ac.uk/software/figtree).

Data availability

The four new DENV-3 genomes obtained from samples collected in Brazil in 2023 are available in GenBank under accession numbers OQ706226-OQ706228 (Roraima State 2023) and OQ868517 (Paraná State, imported from Suriname). We also deposited another DENV-3 GIII genome obtained from a sample collected in Amazonas 2006 (OQ727062).

Acknowledgments

We acknowledge all data contributors, i.e., authors and their originating laboratories responsible for obtaining and submitting timely Dengue virus genomes, including metadata, via GenBank or other public databases. We appreciate the support of FIOCRUZ Genomics Surveillance Network members, as well as the Roraima State and Paraná State surveillance teams for the partnership in viral surveillance in Brazil. We would also like to thank Claudia Nunes Duarte dos Santos for critically reading the manuscript. Funding support: FAPEAM (Universal/AM call 2019; Rede Genômica de Vigilância em Saúde - REGESAM); Inova Fiocruz/Fundação Oswaldo Cruz (Inova Amazônia); Departamento de Ciência e Tecnologia (DECIT) of the Brazilian MoH; CNPq through their productivity research fellowships (306146/2017–7, 303902/2019–1 and 304883/2020-4, respectively): F.G.N., G.L.W., and G.B., respectively. FAPERJ (Grant number E-26/202.896/2018).

References

1. Saldanha RF, Bastos RR, Barcellos C. Microdatasus: pacote para download e pré-processamento de microdados do Departamento de Informática do SUS (DATASUS). Cad Saude Publica. 2019;35:e00032419. PubMed https://doi.org/10.1590/0102-311x00032419

2. Katoh K, Standley DM. MAFFT multiple sequence alignment software version 7: improvements in performance and usability. Mol Biol Evol. 2013;30:772–80. PubMed https://doi.org/10.1093/molbev/mst010

3. Minh BQ, Schmidt HA, Chernomor O, Schrempf D, Woodhams MD, von Haeseler A, et al. IQ-TREE 2: new models and efficient methods for phylogenetic inference in the genomic era. Mol Biol Evol. 2020;37:1530–4. PubMed https://doi.org/10.1093/molbev/msaa015

4. Kalyaanamoorthy S, Minh BQ, Wong TKF, von Haeseler A, Jermiin LS. ModelFinder: fast model selection for accurate phylogenetic estimates. Nat Methods. 2017;14:587–9. PubMed https://doi.org/10.1038/nmeth.4285

5. Shimodaira H, Hasegawa M. Multiple comparisons of log-likelihoods with applications to phylogenetic inference. Mol Biol Evol. 1999;16:1114–6. https://doi.org/10.1093/oxfordjournals.molbev.a026201

6. Hoang DT, Chernomor O, von Haeseler A, Minh BQ, Vinh LS. UFBoot2: improving the ultrafast bootstrap approximation. Mol Biol Evol. 2018;35:518–22. PubMed https://doi.org/10.1093/molbev/msx281

7. Suchard MA, Lemey P, Baele G, Ayres DL, Drummond AJ, Rambaut A. Bayesian phylogenetic and phylodynamic data integration using BEAST 1.10. Virus Evol. 2018;4:vey016. PubMed https://doi.org/10.1093/ve/vey016

8. Li W, Godzik A. Cd-hit: a fast program for clustering and comparing large sets of protein or nucleotide sequences. Bioinformatics. 2006;22:1658–9. PubMed https://doi.org/10.1093/bioinformatics/btl158

9. Martin DP, Varsani A, Roumagnac P, Botha G, Maslamoney S, Schwab T, et al. RDP5: a computer program for analyzing recombination in, and removing signals of recombination from, nucleotide sequence datasets. Virus Evol. 2021;7:veaa087. **PMID 33936774**

10. Rambaut A, Lam TT, Max Carvalho L, Pybus OG. Exploring the temporal structure of heterochronous sequences using TempEst (formerly Path-O-Gen). Virus Evol. 2016;2:vew007. PubMed https://doi.org/10.1093/ve/vew007

11. Drummond AJ, Ho SYW, Phillips MJ, Rambaut A. Relaxed phylogenetics and dating with confidence. PLoS Biol. 2006;4:e88. PubMed https://doi.org/10.1371/journal.pbio.0040088

12. Ferreira MAR, Suchard MA. Bayesian analysis of elapsed times in continuous-time Markov chains. Can J Stat Rev Can Stat. 2008;36:355–68. https://doi.org/10.1002/cjs.5550360302

13. Lemey P, Rambaut A, Drummond AJ, Suchard MA. Bayesian phylogeography finds its roots. PLOS Comput Biol. 2009;5:e1000520. https://doi.org/10.1371/journal.pcbi.1000520

14. Rambaut A, Drummond AJ, Xie D, Baele G, Suchard MA. Posterior summarization in Bayesian phylogenetics using Tracer 1.7. Syst Biol. 2018;67:901–4. PubMed https://doi.org/10.1093/sysbio/syy032

Appendix Table 1. Socio-demographic and laboratory characteristics of the DENV-3 cases, Brazil, 2023.

IDs	Municipality	Collection	Age/Sex	Symptoms	Ct	Genome length	GenBank accession
Fiocruz-ILMD-LACENRR-AR2300001	Cantá	2023–03–04	25/F	F, M	31	10,697	OQ706226
Fiocruz-ILMD-LACENRR-AR2300002	Boa Vista	2023–01–22	8/M	F, L, P, PE, T	28	10,511	OQ706227
Fiocruz-ILMD-LACENRR-AR2300003	Boa Vista	2023–01–03	50/F	F, BP, M, R, V	29	10,553	OQ706228
Fiocruz-ILMD-LACENPR-AR2300004	Curitiba*	2023–03–12	32/M	F, Fa, H, M, N, R, V	27	10,673	OQ868517

Symptoms: BP = back pain, F = Fever, Fa = fatigue, H = headache, L = leucopenia, M = myalgia, N = nausea, p = polyarthralgia, PE = periarticular edema, R = rash, T = thrombocytopenia, V = vomiting. * Although the sample was collected in Curitiba, Paraná, Brazil, the patient arrived in Brazil in 2023–03–09 with dengue symptoms, thus it is considered by local authorities as an imported case from Suriname.

Appendix Table 2. DENV-3 genomes

Accession	Country	Collection date
AY099336	Sri Lanka	2000
AY099337	Martinique	1999
AY662691	Singapore	2004
AY679147	Brazil	2002
AY770511	India	2003
DQ675533	Taiwan	1999
EF629366	Brazil	2004-11
EF629367	Brazil	2004-11
EF629368	Brazil	2004-11
EF629369	Brazil	2002-01
EF643017	Brazil	2003
EU081181	Singapore	2004
EU081182	Singapore	2005
EU081183	Singapore	2005
EU081184	Singapore	2005
EU081185	Singapore	2005
EU081186	Singapore	2005
EU081187	Singapore	2005
EU081188	Singapore	2005
EU081189	Singapore	2005
EU081190	Singapore	2005
EU081191	Singapore	2005
EU081192	Singapore	2005
EU081193	Singapore	2005
EU081194	Singapore	2005
EU081195	Singapore	2005
EU081196	Singapore	2005
EU081197	Singapore	2005
EU081198	Singapore	2005
EU081199	Singapore	2005

Accession	Country	Collection date
EU081200	Singapore	2005
EU081201	Singapore	2005
EU081202	Singapore	2005
EU081203	Singapore	2005
EU081204	Singapore	2005
EU081205	Singapore	2005
EU081206	Singapore	2005
EU081207	Singapore	2005
EU081208	Singapore	2005
EU081209	Singapore	2005
EU081210	Singapore	2005
EU081211	Singapore	2005
EU081212	Singapore	2005
EU081213	Singapore	2005
EU081214	Singapore	2005
EU081215	Singapore	2005
EU081216	Singapore	2005
EU081217	Singapore	2005
EU081218	Singapore	2005
EU081219	Singapore	2005
EU081220	Singapore	2005
EU081222	Singapore	2005
EU081224	Singapore	2005
EU081225	Singapore	2005
EU482555	USA	2006
EU482558	USA	1998
EU482559	USA	1998
EU482563	USA	1998
EU482564	USA	2003
EU482566	USA	1998
EU482595	USA	2003
EU482596	USA	1998
EU482612	Venezuela	2001
EU482613	Venezuela	2001
EU482614	Venezuela	2001
EU529683	Venezuela	2007
EU529684	Venezuela	2001
EU529685	Venezuela	2001
EU529686	Venezuela	2001
EU529687	Venezuela	2001
EU529688	Venezuela	2001
EU529689	Venezuela	2001
EU529690	Venezuela	2001
EU529691	Venezuela	2001
EU529692	USA	2006
EU529696	USA	1999
EU529697	USA	2000
EU529698	USA	2006
EU529699	USA	2006
EU529702	USA	2003
EU529703	USA	1998
EU529704	USA	2004
EU529705	USA	2004
EU569688	Venezuela	2001
EU569689	Venezuela	2001
EU569690	Venezuela	2001
EU569691	Venezuela	2001
EU596492	USA	2007
EU596493	USA	2007
EU596494	USA	2007
EU660420	Venezuela	2001
EU687196	USA	2002
EU687197	USA	2003
EU687198	USA	2003
EU687218	USA	1998

Accession	Country	Collection date
EU687219	USA	1999
EU687221	USA	2000
EU687226	USA	1999
EU687233	USA	2002
EU687234	USA	2002
EU687239	USA	2003
EU726768	USA	2000
EU726769	USA	2003
EU726771	USA	1998
EU726772	USA	1998
EU726773	USA	1999
EU726774	USA	1999
EU781136	USA	1999
EU781137	USA	1999
EU854291	Venezuela	2004
EU854292	Venezuela	2005
EU854298	USA	2002
EU932687	Venezuela	2007
EU932688	Venezuela	2007
FJ024465	USA	2004
FJ024466	USA	2004
FJ024467	USA	2004
FJ024468	USA	2004
FJ024469	USA	2004
FJ024470	USA	2004
FJ024471	USA	2004
FJ177308	Brazil	2001
FJ182004	USA	2004
FJ182005	USA	2004
FJ182006	USA	2004
FJ182007	USA	2005
FJ182008	USA	2005
FJ182009	USA	2005
FJ182010	USA	2005
FJ182011	USA	2005
FJ182013	USA	1998
FJ182015	Venezuela	2001
FJ182037	USA	2005
FJ182038	USA	2005
FJ182039	USA	2005
FJ182040	USA	2005
FJ182041	USA	2005
FJ205870	USA	2003
FJ205871	USA	1999
FJ373302	USA	2004
FJ373303	Venezuela	2001
FJ373304	Venezuela	2004
FJ373306	USA	2002
FJ390371	USA	2003
FJ390372	USA	2003
FJ390373	USA	2002
FJ390375	USA	1999
FJ390376	USA	1999
FJ390377	USA	1999
FJ410176	USA	2000
FJ410177	USA	2000
FJ410178	USA	2002
FJ478456	USA	2002
FJ547069	USA	1999
FJ547070	USA	1998
FJ547071	USA	2000
FJ547072	USA	2000
FJ547073	USA	2000
FJ547074	USA	2000
FJ547075	USA	2000

Accession	Country	Collection date
FJ547076	USA	2000
FJ547077	USA	2000
FJ547078	USA	2000
FJ547079	USA	2001
FJ547080	USA	2001
FJ547081	USA	2001
FJ547082	USA	2001
FJ547083	USA	2002
FJ547084	USA	2002
FJ547085	USA	2006
FJ562107	USA	2000
FJ639746	Venezuela	2000
FJ639747	Venezuela	2000
FJ639749	Venezuela	2000
FJ639750	Venezuela	2000
FJ639751	Venezuela	2001
FJ639752	Venezuela	2001
FJ639753	Venezuela	2001
FJ639754	Venezuela	2001
FJ639755	Venezuela	2001
FJ639756	Venezuela	2001
FJ639757	Venezuela	2001
FJ639758	Venezuela	2001
FJ639759	Venezuela	2001
FJ639760	Venezuela	2001
FJ639761	Venezuela	2001
FJ639762	Venezuela	2001
FJ639763	Venezuela	2001
FJ639765	Venezuela	2001
FJ639766	Venezuela	2001
FJ639767	Venezuela	2001
FJ639768	Venezuela	2001
FJ639769	Venezuela	2001
FJ639770	Venezuela	2001
FJ639771	Venezuela	2001
FJ639772	Venezuela	2007
FJ639774	Venezuela	2001
FJ639775	Venezuela	2002
FJ639776	Venezuela	2002
FJ639777	Venezuela	2002
FJ639778	Venezuela	2002
FJ639779	Venezuela	2003
FJ639780	Venezuela	2003
FJ639781	Venezuela	2003
FJ639782	Venezuela	2003
FJ639784	Venezuela	2003
FJ639785	Venezuela	2003
FJ639786	Venezuela	2003
FJ639787	Venezuela	2004
FJ639789	Venezuela	2004
FJ639790	Venezuela	2004
FJ639791	Venezuela	2004
FJ639792	Venezuela	2004
FJ639793	Venezuela	2004
FJ639795	Venezuela	2004
FJ639798	Venezuela	2004
FJ639799	Venezuela	2004
FJ639800	Venezuela	2004
FJ639801	Venezuela	2004
FJ639803	Venezuela	2005
FJ639804	Venezuela	2005
FJ639805	Venezuela	2005
FJ639807	Venezuela	2005
FJ639810	Venezuela	2005
FJ639816	Venezuela	2005

Accession	Country	Collection date
FJ639817	Venezuela	2006
FJ639825	Venezuela	2006
FJ639826	Venezuela	2008
FJ639827	Venezuela	2008
FJ644564	India	2007
FJ744700	Venezuela	2001
FJ810416	Venezuela	2001
FJ850048	Nicaragua	2008
FJ850049	Nicaragua	2008
FJ850052	Nicaragua	2008
FJ850055	USA	2004
FJ850056	USA	2004
FJ850079	Brazil	2003
FJ850080	Brazil	2003
FJ850083	Brazil	2004
FJ850086	Brazil	2005
FJ850089	Brazil	2006
FJ850092	Brazil	2007
FJ850094	Brazil	2008
FJ850096	Venezuela	2001
FJ850097	Venezuela	2001
FJ850098	Venezuela	2001
FJ850109	Venezuela	2007
FJ850110	Venezuela	2007
FJ850111	Venezuela	2007
FJ873812	Nicaragua	2008
FJ873813	Nicaragua	2008
FJ882571	SriLanka	1989
FJ882572	SriLanka	1989
FJ882573	SriLanka	1993
FJ882574	SriLanka	1985
FJ882575	Mozambique	1985
FJ882576	Nicaragua	1994
FJ882577	Venezuela	2001
FJ882578	Venezuela	2001
FJ898440	Mexico	2003
FJ898441	Mexico	2006
FJ898442	Mexico	2007
FJ898443	Colombia	2003
FJ898444	Colombia	2005
FJ898445	Colombia	2007
FJ898446	Brazil	2001
FJ898447	Brazil	2003
FJ898457	Ecuador	2000
FJ898458	Peru	2002
FJ898459	Trinidad and Tobago	2002
FJ898462	Anguilla	2001
FJ898463	Saint Lucia	2001
FJ898464	Guyana	2002
FJ898468	Venezuela	2000
FJ898469	Venezuela	2001
FJ898470	Venezuela	2001
FJ898471	Venezuela	2002
FJ898472	Venezuela	2003
FJ898473	Venezuela	2003
FJ898474	Venezuela	2007
FJ898475	Nicaragua	2008
FJ898476	Nicaragua	2008
FJ913015	Brazil	2001
GQ199860	Nicaragua	2008
GQ199861	Nicaragua	2008
GQ199862	Nicaragua	2008
GQ199863	Nicaragua	2008
GQ199864	Nicaragua	2008
GQ199865	Nicaragua	2009

Accession	Country	Collection date
GQ199870	Nicaragua	2008
GQ199871	Nicaragua	2008
GQ199886	Nicaragua	1998
GQ199887	Sri Lanka	1983
GQ199888	Sri Lanka	1983
GQ199889	Sri Lanka	1983
GQ199891	Colombia	2001
GQ252674	Sri Lanka	1997
GQ252678	Venezuela	2001
GQ466079	India	2008
GQ868546	Brazil	2006
GQ868547	Brazil	2006
GQ868548	Brazil	2006
GQ868571	Colombia	2002
GQ868572	Colombia	2003
GQ868573	Colombia	2003
GQ868574	Colombia	2003
GQ868575	Colombia	2004
GQ868576	Colombia	2005
GQ868577	Colombia	2005
GQ868578	Colombia	2007
GQ868586	Venezuela	2007
GQ868587	Venezuela	2007
GQ868616	Saint Lucia	2001
GQ868617	Trinidad and Tobago	2002
GU131844	Brazil	2006
GU131845	Brazil	2006
GU131846	Brazil	2006
GU131847	Brazil	2006
GU131848	Brazil	2006
GU131849	Brazil	2006
GU131850	Brazil	2006
GU131851	Brazil	2006
GU131852	Brazil	2006
GU131853	Brazil	2006
GU131854	Brazil	2006
GU131855	Brazil	2006
GU131856	Brazil	2006
GU131857	Brazil	2006
GU131858	Brazil	2006
GU131859	Brazil	2006
GU131860	Brazil	2006
GU131861	Brazil	2007
GU131862	Brazil	2007
GU131865	Brazil	2006
GU131866	Brazil	2007
GU131867	Brazil	2007
GU131868	Brazil	2007
GU131869	Brazil	2007
GU131870	Brazil	2007
GU131871	Brazil	2007
GU131872	Brazil	2007
GU131873	Brazil	2007
GU131874	Brazil	2007
GU131875	Brazil	2007
GU131876	Brazil	2007
GU131877	Brazil	2007
GU131878	Brazil	2007
GU131950	Colombia	2001
GU131951	Colombia	2003
GU131952	Colombia	2003
GU131953	Colombia	2004
GU131954	Colombia	2006
GU363549	China	2009-08-06
GU370053	Singapore	2007-05

Accession	Country	Collection date
HM181972	Nicaragua	2009
HM181973	Nicaragua	2009
HM181974	Nicaragua	2009
HM181975	Nicaragua	2009
HM181976	Nicaragua	2009
HM181977	Nicaragua	2009
HM181978	Nicaragua	2009
HM631856	Nicaragua	2009
HM631857	Nicaragua	2009
HM631858	Nicaragua	2009
HM631859	Nicaragua	2009
HM631860	Nicaragua	2009
HM631861	Nicaragua	2009
HM631862	Nicaragua	2009
HM631863	Nicaragua	2009
HM631864	Nicaragua	2009
HM631869	Nicaragua	2009
HM756274	Nicaragua	2009
HM756275	Nicaragua	2009
HM756276	Nicaragua	2009
HM756277	Nicaragua	2009
HM756278	Nicaragua	2009
HM756279	Nicaragua	2009
HM756280	Nicaragua	2009
HM756281	Nicaragua	2009
HM756282	Nicaragua	2009
HQ166030	Nicaragua	2009
HQ166031	Nicaragua	2009
HQ166032	Nicaragua	2009
HQ166033	Nicaragua	2009
HQ166034	Nicaragua	2009
HQ235027	Paraguay	2007
HQ332170	Venezuela	2006
HQ332171	Venezuela	2006
HQ541785	Nicaragua	2009
HQ541789	Nicaragua	2008
HQ541790	Nicaragua	2008
HQ541791	Nicaragua	2008
HQ541795	Nicaragua	2009
HQ541796	Nicaragua	2009
HQ541797	Nicaragua	2009
HQ541802	Nicaragua	2009
HQ541804	Nicaragua	2009
HQ541806	Nicaragua	2009
HQ671176	Nicaragua	2009
HQ671177	Nicaragua	2009
HQ671178	Nicaragua	2009
HQ705609	Nicaragua	2009
HQ705610	Nicaragua	2009
HQ705611	Nicaragua	2009
HQ705612	Nicaragua	2009
HQ705613	Nicaragua	2009
HQ705614	Nicaragua	2009
HQ705615	Nicaragua	2009
HQ705616	Nicaragua	2009
HQ705617	Nicaragua	2009
HQ705618	Nicaragua	2009
HQ705619	Nicaragua	2009
HQ705620	Nicaragua	2009
HQ705621	Nicaragua	2009
HQ705622	Nicaragua	2009
HQ705623	Nicaragua	2009
HQ891025	Nicaragua	2009
JF504679	China	2009-09
JF808118	Brazil	2002

Accession	Country	Collection date
JF808119	Brazil	2004
JF808120	Brazil	2009
JF808121	Brazil	2007
JF808122	Paraguay	2003
JF808123	Paraguay	2002
JF808124	Brazil	2003
JF808125	Brazil	2002
JF808126	Brazil	2003
JF808127	Brazil	2002
JF808128	Paraguay	2003
JF808129	Paraguay	2003
JF920393	Nicaragua	2009
JF920394	Nicaragua	2009
JF920395	Nicaragua	2009
JF920396	Nicaragua	2009
JF920397	Nicaragua	2010
JF920398	Nicaragua	2009
JF920399	Nicaragua	2009
JF920400	Nicaragua	2009
JF920401	Nicaragua	2010
JF920402	Nicaragua	2009
JF920403	Nicaragua	2009
JF920404	Nicaragua	2009
JF920405	Nicaragua	2010
JF920406	Nicaragua	2010
JF920407	Nicaragua	2009
JF920408	Nicaragua	2010
JF920409	Nicaragua	2010
JF937620	Nicaragua	2010
JF937621	Nicaragua	2010
JF937622	Nicaragua	2010
JF937623	Nicaragua	2010
JF937624	Nicaragua	2010
JF937625	Nicaragua	2010
JF937626	Nicaragua	2010
JF937627	Nicaragua	2010
JF937628	Nicaragua	2010
JF937629	Nicaragua	2010
JF937630	Nicaragua	2009
JF937631	Nicaragua	2009
JF937632	Nicaragua	2009
JF937633	Nicaragua	2010
JF937634	Nicaragua	2010
JF937636	Nicaragua	2009
JF937637	Nicaragua	2010
JF937638	Nicaragua	2010
JF937639	Nicaragua	2010
JF937640	Nicaragua	2010
JF937641	Nicaragua	2009
JF937642	Nicaragua	2009
JF937643	Nicaragua	2009
JF937646	Nicaragua	2010
JF937647	Nicaragua	2009
JF937648	Nicaragua	2010
JF937652	Nicaragua	2009
JN000936	Nicaragua	2009
JN000937	Nicaragua	2010
JN000938	Nicaragua	2010
JN093513	Nicaragua	2010
JN093514	Nicaragua	2009
JN093515	Nicaragua	2010
JN093517	Nicaragua	2009
JN183884	Nicaragua	2009
JN662391	China	2009-08-06
JQ411814	Sri Lanka	1989

Accession	Country	Collection date
JQ922555	India	1966
JQ922556	India	2005
JQ922557	India	2005
JX669489	Brazil	2003
JX669490	Brazil	2002
JX669491	Brazil	2002
JX669492	Brazil	2005
JX669493	Brazil	2005
JX669494	Brazil	2005
JX669495	Brazil	2004
JX669496	Brazil	2006
JX669497	Brazil	2005
JX669498	Brazil	2004
JX669499	Brazil	2004
JX669500	Brazil	2005
JX669501	Brazil	2005
JX669502	Brazil	2005
JX669503	Brazil	2005
JX669504	Brazil	2006
JX669505	Brazil	2006
JX669506	Brazil	2006
JX669507	Brazil	2006
JX669508	Brazil	2006
KC425219	Brazil	2002
KF041254	Pakistan	2008
KF041255	Pakistan	2007
KF041256	Pakistan	2006
KF041257	Pakistan	2006
KF041258	Pakistan	2009
KF041259	Pakistan	2006
KF921913	Nicaragua	2009
KF921914	Nicaragua	2009
KF921916	Nicaragua	2009
KF921920	Nicaragua	2009
KF921921	Nicaragua	2009
KF921922	Nicaragua	2009
KF921923	Nicaragua	2009
KF921924	Nicaragua	2010
KF921926	Nicaragua	2010
KF921928	Nicaragua	2010
KF921929	Nicaragua	2010
KF954945	China	2013-08-08
KF954946	China	2013-08-08
KF954947	China	2013-08-08
KF954948	China	2013-08-08
KF954949	China	2013-08-08
KF955449	Venezuela	2001-10-13
KF955451	Venezuela	2003-12-01
KF955453	Venezuela	2003-12-19
KF955454	Venezuela	2004-11-07
KF955456	Puerto Rico	2006
KF955465	Puerto Rico	2000
KF955466	Puerto Rico	2000
KF955468	Puerto Rico	2001
KF955471	Venezuela	2004
KF955472	Venezuela	2004
KF955473	Brazil	2002
KF955474	Sri Lanka	1989
KF955479	Venezuela	2001
KF955481	Venezuela	2007
KF955486	Venezuela	2001
KF955487	Venezuela	2001
KF955490	Nicaragua	2008
KF955505	Grenada	2002
KF971695	Nicaragua	2009

Accession	Country	Collection date
KF971709	Nicaragua	2009
KF973476	Nicaragua	2011
KF973477	Nicaragua	2011
KF973478	Nicaragua	2012
KF973479	Nicaragua	2012
KF973480	Nicaragua	2012
KF973481	Nicaragua	2011
KF973482	Nicaragua	2011
KF973483	Nicaragua	2011
KF973484	Nicaragua	2011
KF973485	Nicaragua	2011
KF973486	Nicaragua	2012
KF973487	Nicaragua	2011
KJ189255	Peru	2002
KJ189256	Peru	2002
KJ189257	Peru	2004
KJ189258	Peru	2002
KJ189259	Peru	2002
KJ189260	Peru	2002
KJ189261	Peru	2008
KJ189262	Peru	2004
KJ189263	Peru	2004
KJ189264	Peru	2004
KJ189265	Peru	2004
KJ189266	Peru	2004
KJ189267	Peru	2007
KJ189268	Peru	2007
KJ189269	Peru	2007
KJ189270	Peru	2007
KJ189271	Peru	2007
KJ189272	Peru	2007
KJ189273	Peru	2007
KJ189274	Peru	2007
KJ189275	Peru	2007
KJ189276	Peru	2007
KJ189277	Peru	2007
KJ189278	Peru	2007
KJ189279	Peru	2007
KJ189280	Peru	2007
KJ189281	Peru	2007
KJ189282	Peru	2007
KJ189283	Peru	2007
KJ189284	Peru	2008
KJ189285	Peru	2008
KJ189286	Peru	2008
KJ189287	Peru	2008
KJ189288	Peru	2008
KJ189289	Peru	2008
KJ189290	Peru	2008
KJ189291	Peru	2008
KJ189292	Peru	2009
KJ189293	Peru	2005
KJ189294	Peru	2006
KJ189295	Peru	2006
KJ189296	Peru	2006
KJ189297	Peru	2006
KJ189298	Peru	2008
KJ189299	Peru	2005
KJ189300	Peru	2008
KJ189301	Peru	2008
KJ643590	Peru	2007
KJ830751	Saudi Arabia	2014-01-26
KT726340	Cuba	2001
KT726341	Cuba	2001
KT726342	Cuba	2001

Accession	Country	Collection date
KT726343	Cuba	2001
KT726344	Cuba	2001
KT726345	Cuba	2002
KT726346	Cuba	2002
KT726347	Cuba	2002
KT726348	Cuba	2002
KT726349	Cuba	2001
KT726350	Cuba	2001
KT726351	Cuba	2001
KT726352	Cuba	2001
KT726353	Cuba	2001
KT726354	Cuba	2001
KT726355	Cuba	2001
KT726356	Cuba	2001
KT726357	Cuba	2001
KT726358	Cuba	2001
KT726359	Cuba	2001
KT726360	Cuba	2001
KT726361	Cuba	2002
KU216208	India	2013-11-11
KU216209	India	2013-11-11
KU509278	Barbados	2007
KU509281	India	2009
KU509282	Senegal	2009
KU509283	Sri Lanka	2006
KU509286	India	2011
KX380841	Singapore	2012
KX380842	Singapore	2013
KX855927	India	2014-10-15
KY921907	Singapore	2015-04
LC379193	Gabon	2016-05-15
LC379194	Gabon	2016-05-20
LC379195	Gabon	2016-07-14
LC379196	Gabon	2016-07-22
LC379197	Gabon	2017-04-15
LC410192	Thailand	2016-10
LC410193	Thailand	2016-12
LC410194	Thailand	2016-12
LC410195	Thailand	2017-01
LT898451	Malaysia	2011
LT898452	Malaysia	2011
LT996904	Malaysia	2007
LT996905	Malaysia	2008
LT996906	Malaysia	2008
LT996907	Malaysia	2010
LT996908	Malaysia	2010
LT996909	Malaysia	2010
LT996910	Malaysia	2010
LT996911	Malaysia	2010
LT996912	Malaysia	1987
MF142763	Thailand	2015-09
MF370226	China	2013-08-20
MG721059	India	2016
MG721061	India	2016
MG721064	India	2016
MH048677	Malaysia	2014-12
MH051731	Malaysia	2014-12
MH051733	Malaysia	2014-12
MH544647	Colombia	2015-08-23
MH544649	Colombia	2015-09-15
MH544650	Colombia	2015-09-07
MH544651	Colombia	2016-04-16
MH822957	India	2013
MH888333	Bolivia	2011-01-01
MH891766	India	2017-02-21

Accession	Country	Collection date
MK858149	India	2016-08-26
MK858150	India	2016-08-10
MK858151	India	2016-11-08
MK858152	India	2016-08-24
MK858153	India	2016-10-14
MK858154	India	2017-10-12
MK858155	India	2017-10-09
MK894339	China	2018-04-09
MK894340	China	2018-10-16
MK894341	China	2018-12-30
MN018367	China	2015-09-13
MN018368	China	2013-07-25
MN018371	China	2015-08-08
MN018372	China	2016-03-18
MN018375	China	2015-10-23
MN018376	China	2015-07-06
MN018378	China	2015-09-30
MN018381	China	2016-05-06
MN018385	China	2016-09-20
MN018386	China	2013-07-28
MN227697	China	2019-05-19
MN227698	China	2019-07-02
MN227699	China	2019-06-30
MN227700	China	2019-07-08
MN227701	China	2019-07-08
MN227702	China	2019-06-01
MN227703	China	2019-07-18
MN253124	India	2017-08-10
MN253125	India	2017-10-27
MN253126	India	2016
MN253127	India	2018-09-06
MN253128	India	2018-09-18
MN253129	India	2018-09-20
MN253130	India	2018-12-09
MN253131	India	2018-12-09
MN253132	India	2018-09-18
MN253133	India	2018-10-13
MN448966	Thailand	2012-09-25
MN448967	Thailand	2012-09-25
MN448986	Thailand	2011-06-14
MN448991	Thailand	2012-05-11
MN448992	Thailand	2012-10-01
MN453624	Singapore	2016-01-20
MN922036	China	2019-07-17
MN964273	China	2019-11-09
MN964274	China	2019-11-21
MT261972	Burkina Faso	2017-10-16
MT261973	Burkina Faso	2017-11-09
MT261974	Burkina Faso	2017-11-13
MT261975	Burkina Faso	2017-11-17
MT261976	Burkina Faso	2017-10-04
MT261977	Burkina Faso	2017-10-12
MT261978	Burkina Faso	2017-10-27
MT261979	Burkina Faso	2017-11-02
MW192820	India	2016-08
MW192821	India	2016-08
MW192822	India	2016-08
MW192823	India	2016-07
MW192824	India	2017-08
MW288025	Senegal	2018-10
MW288026	Senegal	2018-10
MW288027	Senegal	2018-10
MW288028	Senegal	2018-10
MW288031	Senegal	2018-11
MW288033	Senegal	2018-11

Accession	Country	Collection date
MW288035	Senegal	2018-11
MW288037	Senegal	2018-11
MW288038	Senegal	2018-11
MW288039	Senegal	2018-11
MW288040	Senegal	2018-11
MW720883	China	2019-09
MW720884	China	2019-09
MW720885	China	2019-09
MW720886	China	2019-09
MW720887	China	2019-09
MW720888	China	2019-09
MW788883	Myanmar	2017-06
MW788884	Myanmar	2017-07
MW788886	Myanmar	2017-07
MW788887	Myanmar	2017-07
MW788888	Myanmar	2017-07
MW788889	Myanmar	2018-07
MW788891	Myanmar	2019-12
MW788893	Myanmar	2019-07
MW788896	Myanmar	2019-05
MW788904	Myanmar	2018-07
MW788906	Myanmar	2018-06
MW788907	Myanmar	2018-10
MW788910	Myanmar	2018-08
MW788911	Myanmar	2018-07
MW945428	Puerto Rico	2006
MW946612	Thailand	2010
MW946615	Thailand	2014
MW946633	Thailand	2013
MW946659	Thailand	2013
MW946662	Thailand	2013
MW946679	Thailand	2014
MW946690	Thailand	2014
MW946693	Thailand	2012
MW946694	Thailand	2014
MW946704	Thailand	2013
MW946711	Thailand	2012
MW946721	Thailand	2011
MW946741	Thailand	2014
MW946763	Thailand	2013
MW946767	Thailand	2006
MW946775	Thailand	2014
MW946778	Thailand	2013
MW946783	Thailand	2012
MW946798	Thailand	2013
MW946799	Thailand	2012
MW946800	Thailand	2012
MW946801	Thailand	2014
MW946802	Thailand	2014
MW946808	Thailand	2010
MW946810	Thailand	2012
MW946815	Thailand	2006
MW946839	Thailand	2011
MW946841	Thailand	2012
MW946843	Thailand	2014
MW946870	Thailand	2014
MW946872	Thailand	2014
MW946881	Thailand	2014
MW946891	Thailand	2011
MW946918	Nicaragua	2009
MW946957	Thailand	2013
MW946958	Thailand	2002
MW946973	Thailand	2013
MW946974	Thailand	2011
MW946979	Thailand	2014

Accession	Country	Collection date
MZ008468	Nicaragua	2014
MZ008469	Nicaragua	2013
MZ008470	Nicaragua	2013
MZ008471	Nicaragua	2013
MZ008472	Nicaragua	2013
MZ008473	Nicaragua	2013
MZ008474	Nicaragua	2013
MZ008475	Nicaragua	2013
MZ008476	Nicaragua	2014
MZ008477	Nicaragua	2013
MZ008478	Nicaragua	2013
MZ312921	India	2018-07-09
MZ544585	Kenya	2019-03
MZ544586	Kenya	2019-03
MZ544587	Kenya	2019-03
MZ544588	Kenya	2019-03
MZ857204	Kenya	2011
MZ857217	Saudi Arabia	2016
OK605762	Paraguay	2007
OK605765	Ecuador	2001
OK605766	Somalia	1993
OM368353	China	2019-05-17
OM417340	Mexico	2021-09-21
OM417341	Mexico	2021-09-19
OM638675	India	2021-11-29
OM865777	Bhutan	2019-07-29
OM865778	Bhutan	2019-07-29
OM865779	Bhutan	2019-07-29
OM865780	Bhutan	2019-07-30
OM865781	Bhutan	2019-07-31
OM865782	Bhutan	2019-08-03
OM865783	Bhutan	2019-08-02
OM865784	Bhutan	2019-08-08
OM865785	Bhutan	2019-08-08
OM865786	Bhutan	2019-08-03
OM865787	Bhutan	2019-08-06
OM865788	Bhutan	2019-08-09
OM865789	Bhutan	2019-08-07
OM865790	Bhutan	2019-08-09
OM865791	Bhutan	2019-08-10
OM865792	Bhutan	2019-08
OM865793	Bhutan	2019-07-19
OM865794	Bhutan	2019-07-27
OM865795	Bhutan	2019-08-24
OM865796	Bhutan	2019-08-20
OM865797	Bhutan	2019-08-20
OM865798	Bhutan	2019-08-22
OM865799	Bhutan	2019-08-19
OM865800	Bhutan	2019-08-19
OM865801	Bhutan	2019-08-19
OM865802	Bhutan	2019-07-17
OM865803	Bhutan	2019-07-17
OM865804	Bhutan	2019-07-28
OM865805	Bhutan	2019-07-18
OM865806	Bhutan	2019-08-26
OM865807	Bhutan	2019-08-24
OM865808	Bhutan	2019-08-28
OM865809	Bhutan	2019-09-04
OM865810	Bhutan	2019-09-02
OM865811	Bhutan	2019-08-31
OM865812	Bhutan	2019-09-07
OM865813	Bhutan	2019-09-09
OM865814	Bhutan	2019-09-09
OM865815	Bhutan	2019-09-10
OM865816	Bhutan	2019-09-14

Accession	Country	Collection date
OM865817	Bhutan	2019-09-21
OM865818	Bhutan	2019-08
OM865819	Bhutan	2019-08
OM865820	Bhutan	2019-08
ON007080	Thailand	2011-08-04
ON055565	Thailand	2010-02-16
ON055567	Thailand	2011-08-25
ON109599	India	2021
ON123655	India	2020
ON123658	India	2019
ON123659	India	2018
ON123660	India	2018
ON123662	India	2018
ON123665	India	2018
ON123669	India	2018
ON123670	India	2018
ON799401	India	2018
ON890788	Ethiopia	2019-11-09
ON890789	Maldives	2019-05-19
ON890819	China	2019-07-08
ON891145	China	2019-06-01
ON900159	China	2019-06-01
ON907582	China	2019-07-08
ON908232	Ethiopia	2019-11-09
ON908233	Maldives	2019-05-19
ON908234	China	2019-07-08
ON908245	China	2019-06-01
OP410993	Singapore	2008-09
OP410994	Singapore	2013-01
OP410997	Singapore	2019-01
OP410998	Singapore	2018-02
OP895705	India	2018
OP895928	Maldives	2019-03
OP895929	Maldives	2019-12
OP921002	India	2022-07-27
OQ339138	India	2022
OQ445895	Cuba	2022-08-01
OQ445896	Cuba	2022-07-25
OQ445897	Cuba	2022-07-25
OQ445898	Cuba	2022-07-30
OQ445899	Cuba	2022-07-28
OQ445901	Cuba	2022-07-25
OQ445902	Cuba	2022-07-26
OQ445903	Cuba	2022-07-19
OQ445904	Cuba	2022-07-30
OQ445905	Cuba	2022-08-02
OQ445906	Cuba	2022-08-03
OQ445907	Cuba	2022-08-03
OQ445908	Cuba	2022-07-27
OQ445909	Cuba	2022-08-08
OQ445910	Cuba	2022-08-08
OQ445911	Cuba	2022-08-06
OQ445912	Cuba	2022-08-09
OQ445913	Cuba	2022-08-09
OQ445914	Cuba	2022-08-04
OQ445915	Cuba	2022-08-10
OQ445916	Cuba	2022-08-10
OQ445917	Cuba	2022-08-08
OQ445918	USA	2022-08-09
OQ445919	USA	2022-08-15
OQ445920	USA	2022-08-18
OQ445921	USA	2022-08-28
OQ445922	USA	2022-08-29
OQ445923	USA	2022-09-05
OQ445924	Cuba	2022-09-02

Accession	Country	Collection date
OQ445925	USA	2022-09-14
OQ445926	Cuba	2022-09-11
OQ445927	USA	2022-09-25
OQ445928	Cuba	2022-09-20
OQ445929	USA	2022-09-25
OQ445930	USA	2022-10-10
OQ445931	USA	2022-10-19
OQ445932	USA	2022-10-23
OQ445933	USA	2022-10-24
OQ445934	USA	2022-10-20
OQ445935	USA	2022-10-26
OQ445936	Cuba	2022-07-26
OQ445937	Cuba	2022-07-22
OQ445938	Cuba	2022-07-21
OQ445939	Cuba	2022-07-22
OQ445940	Cuba	2022-07-09
OQ445941	Cuba	2022-07-21
OQ445942	Cuba	2022-07-22
OQ445943	Cuba	2022-07-19
OQ445944	Cuba	2022-07-22
OQ445945	Cuba	2022-07-17
OQ445946	USA	2022-08-09
OQ445947	USA	2022-08-05
OQ445948	USA	2022-07-29
OQ445949	Cuba	2022-07-15
OQ445950	Cuba	2022-07-09
OQ445951	Cuba	2022-07-09
OQ445952	Cuba	2022-07-10
OQ445953	USA	2022-07-13
OQ445954	Cuba	2022-07-08
OQ445955	Cuba	2022-07-09
OQ445956	Cuba	2022-07-06
OQ445957	Cuba	2022-06-25
OQ445958	Cuba	2022-06-28
OQ445959	Cuba	2022-06-29
OQ445960	Cuba	2022-06-21
OQ445961	Cuba	2022-05-22
OQ445962	Cuba	2022-06-28
OQ836205	Puerto Rico	2022-10-29
OQ836206	Puerto Rico	2022-08-20
OQ836207	Puerto Rico	2022-09-02
OQ836208	Puerto Rico	2022-12-27
OQ836209	Puerto Rico	2023-01-24
OQ836210	Puerto Rico	2022-07-30
OQ836211	Puerto Rico	2022-07-27
OQ727062	Brazil	2006-04-17

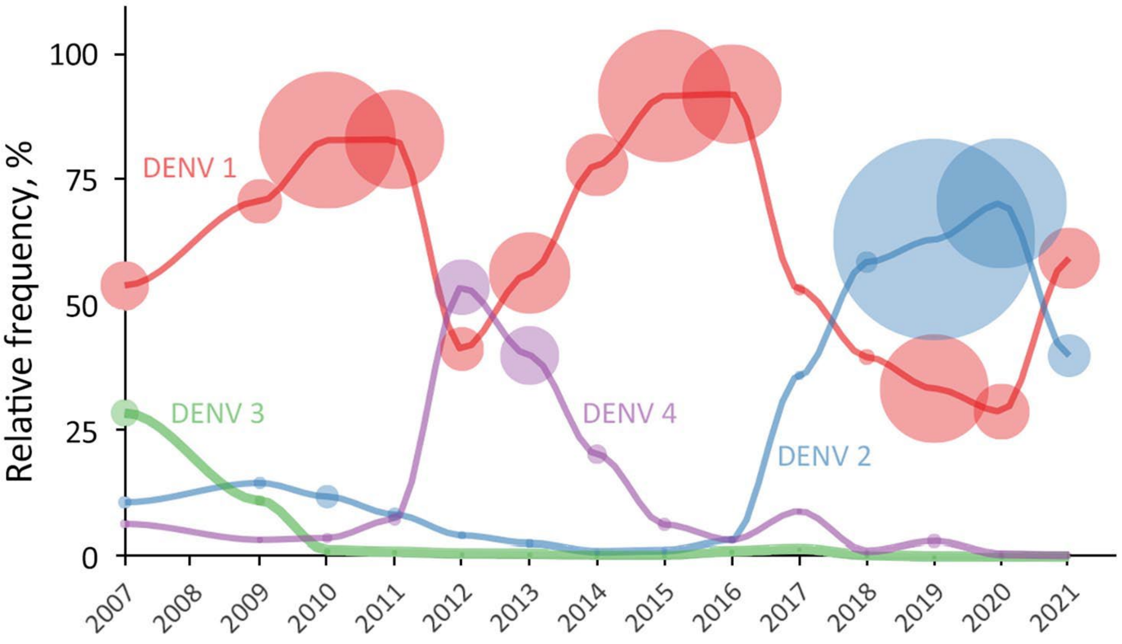

Appendix Figure. Relative frequencies of the four DENV serotypes in Brazil since 2007. The size of the circles is relative to the number of DENV cases attributable to each serotype in each year.
